# Supplementary material for: Causal Relationship between Aging and Anorexia Nervosa: A White-Matter-Microstructure-Mediated Mendelian Randomization Analysis
Source: Biomedicines. 2024 Aug 16;12(8):1874. doi: 10.3390/biomedicines12081874 (PMC11351342; doi:10.3390/biomedicines12081874)
Supplement: Supplementary file 1 [file biomedicines-12-01874-s001.zip › Supplementary Figure/Figure S8/Leaveoneout plot of MR analysis for TL to ubm-b-1649.pdf]

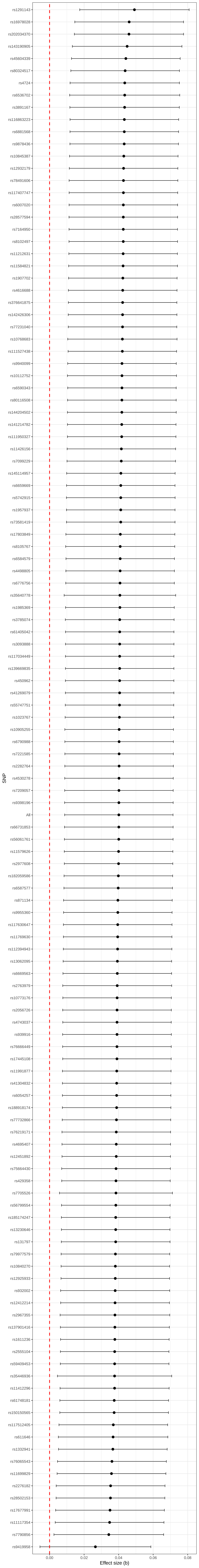

rs1291143
rs16978028
rs202034370
rs143190905
rs45604339
rs80324517
rs4724
rs6536702
rs3891167
rs116863223
rs6881568
rs9878436
rs10845387
rs12932179
rs78491606
rs117407747
rs6007020
rs28577594
rs7164950
rs8102497
rs11212631
rs11584821
rs1907702
rs4616688
rs376641875
rs142426306
rs77231040
rs10768683
rs111527438
rs9940099
rs10112752
rs6590343
rs80116508
rs144204502
rs141214782
rs111950327
rs11426156
rs7099229
rs145114957
rs6659669
rs5742915
rs1957937
rs73581419
rs17803849
rs8105767
rs6584579
rs4498805
rs6776756
rs35640778
rs1985369
rs3785074
rs61405042
rs3093888
rs117034449
rs139669835
rs450962
rs41269079
rs55747751
rs1023767
rs10905255
rs6790988
rs7221585
rs2282764
rs4530278
rs7209057
rs9398196
All
rs66731853
rs56061761
rs11579626
rs2977608
rs182059586
rs6587577
rs871134
rs9955360
rs117630647
rs11769630
rs112394943
rs13062095
rs6669563
rs2763979
rs10773176
rs2056726
rs4743037
rs939916
rs76666449
rs17445108
rs11991877
rs41304832
rs6054257
rs188918174
rs77732866
rs76219171
rs4695407
rs12451892
rs75664430
rs429358
rs7705526
rs56799554
rs185174247
rs13230646
rs131797
rs79977579
rs10840270
rs12925933
rs932002
rs12412214
rs2967355
rs137901416
rs1611236
rs2555104
rs59409453
rs35446936
rs11412296
rs61748181
rs150150565
rs117512405
rs611646
rs1332941
rs76065543
rs11699829
rs2276182
rs28502153
rs17677991
rs11117354
rs7790856
rs9419958
SNP
0.00
0.02
0.04
0.06
0.08
Effect size (b)
